# Anæsthesia by Chemically Pure Ether

**Published:** 1865

**Authors:** 


Anæsthesia by Chemically Pure Ether.—MM. Regnauld and Advian, pharmaceutists, (*Rev. D. Therap. Med. Chir.*) laid before the Imperial Academy of Medicine, Dec. 27, 1864, a work on the method of obtaining chemically pure sulphuric acid. M. Gosselin stated that at the request of MM. R. and A., he had tried their pure sulphuric ether, and found its effects far more rapid and certain than that of ordinary ether, and that the period of excitement did not occur. Four to eight minutes sufficed for the production of complete anæsthesia, and as death had been produced in a certain number of cases from the inhalation of chloroform, whilst none had resulted from ether, he thought the latter should be preferred to the former.

